# In Silico and In Vitro Experimental Studies of New Dibenz[*b*,*e*]oxepin-11(6*H*)one O-(arylcarbamoyl)-oximes Designed as Potential Antimicrobial Agents

**DOI:** 10.3390/molecules25020321

**Published:** 2020-01-13

**Authors:** Ilinca Margareta Vlad, Diana Camelia Nuta, Cornel Chirita, Miron Teodor Caproiu, Constantin Draghici, Florea Dumitrascu, Coralia Bleotu, Speranța Avram, Ana Maria Udrea, Alexandru Vasile Missir, Luminita Gabriela Marutescu, Carmen Limban

**Affiliations:** 1 Department of Pharmaceutical Chemistry, University of Medicine and Pharmacy “Carol Davila”, Traian Vuia 6, sect. 2, 020956 Bucharest, Romania; ilincamargaretavlad@gmail.com (I.M.V.); diananuta@yahoo.com (D.C.N.); missir_alexandru@yahoo.com (A.V.M.); carmen_limban@yahoo.com (C.L.); 2 Department of Pharmacology and Clinical Pharmacy, University of Medicine and Pharmacy “Carol Davila”, Traian Vuia 6, sect. 2, 020956 Bucharest, Romania; chirita.cornel@gmail.com; 3 The Organic Chemistry Center of Romanian Academy “Costin D. Nenitescu”, Splaiul Independenței 202B, 77208 Bucharest, Romania; dorucaproiu@gmail.com (M.T.C.); cst_drag@yahoo.com (C.D.); fdumitra@yahoo.com (F.D.); 4 Ștefan S Nicolau Institute of Virology, Romanian Academy, 285 Mihai Bravu Avenue, 030304 Bucharest, Romania; 5 Research Institute of the University of Bucharest (ICUB) and Microbiology Immunology Department, Faculty of Biology, University of Bucharest, 1-3 Ale. Portocalelor, 060101 Bucharest, Romania; lumi.marutescu@gmail.com; 6 Department of Anatomy, Animal Physiology and Biophysics, Faculty of Biology, University of Bucharest, 1-3 Ale. Portocalelor, 060101 Bucharest, Romania; speranta.avram@gmail.com (S.A.); m.a.u.anamaria@gmail.com (A.M.U.); 7 Laser Department, National Institute for Laser, Plasma and Radiation Physics, Atomistilor Street 409, 077125 Magurele, Romania

**Keywords:** dibenz[*b*,*e*]oxepins, oxime carbamates, antibacterial, antifungal, antibiofilm, in silico, in vitro cytotoxicity

## Abstract

In a drug-repurposing-driven approach for speeding up the development of novel antimicrobial agents, this paper presents for the first time in the scientific literature the synthesis, physico-chemical characterization, in silico analysis, antimicrobial activity against bacterial and fungal strains in planktonic and biofilm growth state, as well as the in vitro cytotoxicity of some new 6,11-dihydrodibenz[*b*,*e*]oxepin-11(6*H*)one O-(arylcarbamoyl)oximes. The structures of intermediary and final substances (compounds **7a**–**j**) were confirmed by ^1^H-NMR, ^13^C-NMR and IR spectra, as well as by elemental analysis. The in silico bioinformatic and cheminformatic studies evidenced an optimal pharmacokinetic profile for the synthesized compounds **7a**–**j**, characterized by an average lipophilic character predicting good cell membrane permeability and intestinal absorption; low maximum tolerated dose for humans; potassium channels encoded by the hERG I and II genes as potential targets and no carcinogenic effects. The obtained compounds exhibited a higher antimicrobial activity against the planktonic Gram-positive *Staphylococcus aureus* and *Bacillus subtilis* strains and the *Candida albicans* fungal strain. The obtained compounds also inhibited the ability of *S. aureus*, *B. subtilis*, *Escherichia coli* and *C. albicans* strains to colonize the inert substratum, accounting for their possible use as antibiofilm agents. All the active compounds exhibited low or acceptable cytotoxicity levels on the HCT8 cells, ensuring the potential use of these compounds for the development of new antimicrobial drugs with minimal side effects on the human cells and tissues.

## 1. Introduction

Antibiotics represent one of the most important therapeutic discoveries in medical history, having contributed to reduce the mortality and morbidity caused by infectious diseases. They are also an essential tool for modern medicine, making possible common life-saving procedures such as transplantation, chemotherapy of cancer and surgery. Unfortunately, antibiotics have been liable to misuse, leading to the emergence and selection of resistant microorganisms. Presently, antibiotic resistance is one of the biggest public health challenges of our time, being responsible each year of at least 2 million cases of infection and at least 23,000 deaths in USA. The CDC AR Threats Report ranks the most dangerous 18 threats (nine Gram-negative, eight Gram-positive bacteria and one fungal species) into three categories based on the level of concern (i.e., urgent, serious, and concerning) about their threat to human health [1]. In some alarming prediction models, although not generally accepted and currently debated [2], the number of deaths caused by AR microorganisms threatens to reach up to 10 million by 2050 and the economic loses associated to AR are estimated at 100 trillion USD [3]. 

The problem of increasing antibiotic resistance is even more threatening when considering the very limited number of new antimicrobial agents that are in development, with only two novel classes—lipopeptides and diarylquinolines, respectively—being introduced after 1980, and the declining interest of pharmaceutical companies for developing novel antibiotics in the last years [4,5,6]. The antibiotic resistance exceeds the borders of healthcare settings, becoming an increasing problem in community-acquired infections and for the environment.

Some scientists are forecasting the beginning of the “post-antibiotic era”, where it will be difficult to effectively control multidrug-resistant infections. Many international authorities advocate for incentives to encourage the development of new antibiotics. In Europe, one of the main aims of the Joint Programming Initiative on Antimicrobial Resistance (JPIAMR) for 2020–2025 is to support innovations for reducing the burden of AR [7].

One safe and successful approach for filling the last decades’ innovation gap and speeding up the discovery of novel antibiotics is repurposing current approved drugs or drugs that previously may have failed in clinical trials, but which have thoroughly studied in terms of toxicity, formulation, and pharmacology [8,9,10]. The key role of different drugs (anti-cancer, antifungal, anti-hyperlipidemia, anti-inflammatory, anti-malarial, anti-parasitic, antivirals, genetic disorder therapeutic agents, immunomodulators etc.) repurposing in the development of antibiotics during 2016–2017 was recently reviewed by Konreddy et al. [10].

One of the various approaches toward developing new classes of antibiotics is focusing on new targets and processes, such as decreasing the pathogenicity of microbial strains by the inhibition of the expression of cell associated and soluble virulence factors. In our previous studies, we have synthesized, characterized and demonstrated that O-acyloximinodibenz[*b*,*e*]oxepins could represent promising candidates for developing new and efficient anti-pathogenic agents [11]. 

Encouraging results of these investigations carried out on dibenz[*b*,*e*]oxepin compounds, and the literature data showing that the carbamoyl and oximino pharmacophore groups are exhibiting anti-infective activity, have led us to combine in a single original molecule such biologically active fragments.

In order to obtain biopharmacophore molecules unquoted in the literature we have conducted the chemical modeling of the position 11 of dibenz[*b*,*e*]oxepinic nucleus by introducing arylcarbamoyloximino side-chains. 

The theoretical premise which could explain the anti-infectious activity of these compounds is the chelating effect of some metallic ions in the carbamoyloximino side chain, the formed compound being able to block the metallic ions of the bacterial enzymes and to disturb the formation of some metaloenzymes.

The dibenz[*b*,*e*]oxepin nucleus represents the basic structure for many drugs, mainly used in psychotropic therapy as antidepressants, anxiolytics, or antipsychotics (doxepin, cidoxepin, spiroxepin, pinoxepin), as analgesics, anti-inflammatory, antipyretics and antiphlogistics (isoxepac, oxepinac), as *β*-sympathomimetics (doxaminol) or as inhibitors of phospholipase A_2_ (olopatadine).

Over the last few years, the dibenz[*b*,*e*]oxepin nucleus has also been used for the design of anti-cancer drugs. The 8-(11-oxo-6,11-dihydro-dibenz[*b*,*e*]oxepin-2-yloxy)-octanoic acid hydroxyamide has been shown to have histone deacetylase (HDAC) inhibitory activity and to induce cancer cells apoptosis [12].

A series of imidazole derivatives of 6,11-dihidrodibenz[*b*,*e*]oxepins were tested for their antibacterial and antifungal activity and proved to be active [13]. Leptosphaerin D, an analogue of arugosin F, is a new polyketide with a dibenz[*b*,*e*]oxepin structure, isolated from solid cultures of *Leptosphaeria* sp., an ascomycete fungus, which showed antimicrobial and antifungal effects against the plant pathogens *Fusarium nivale* and *Piricularia oryzae* [14].

The carbamoyloximino moiety can be found in the structures of antibacterial and antifungal agents. For example, O-substituted thiophene oxime carbamates are used as antibacterial and antifungal agents, being active against *Staphylococcus aureus* and *Chaetomium globosum* [15]. New N-methyl-N-(3-trifluoromethylphenylsulfenyl)-carbonyloxime carbamates display strong insecticidal, acaricidal, nematicidal, fungicidal and bactericidal properties [16]. Tricyclic compounds such as the 2-adamantanone oxime carbamates showed in vitro antifungal activity against yeast and systemic mycoses and dermatophytes [17]. 2-Bromo-6-(4-pyridin-2-yl-piperazin-1-yl)-indeno [2,1-*c*]quinolin-7-one *N*,*N*-dimethylcarbamoyl oxime showed tuberculostatic activity, being active against *Mycobacterium tuberculosis* H37Rv [18]. In fact, this structural feature is found in compounds used as acaricides, insecticides and/or nematocides (aldicarb, aldoxycarb, oxamyl alanycarb, tirpate) and in tertiary butyl-substituted carbamoyl oximes with the same actions [19]. The carbamoyloximino moiety present in such molecules contributes to the improvement of their pharmacological and pharmacokinetic properties [20].

This paper presents the synthesis, physico-chemical characterization, in silico analysis, antimicrobial activity against bacterial and fungal strains in planktonic and biofilm growth state, as well as the in vitro cytotoxicity of new 6,11-dihydro-dibenz[*b*,*e*]oxepin-11(6*H*)one O-(arylcarbamoyl)oximes.

## 2. Results

### 2.1. Chemistry and Spectral Data

In our synthesis we used 6,11-dihydro-dibenz[*b*,*e*]oxepin-11(6H)-one (**1a**), and its 2-methyl- (**1b**) and 2-ethyl- (**1c**) derivatives, as intermediate substances. The mentioned ketones were obtained after cyclization of 2-(phenoxymethyl)benzoic acid (**2a**), 2-(4-methylphenoxymethyl)benzoic acid (**2b**), and 2-(4-ethylphenoxymethyl)benzoic acid (**2c**).

The acids **2a**, **2b** and **2c** resulted by phtalide (**3**) condensation with a suspension of potassium phenoxide, potassium *p*-cresolate or potassium *p*-ethylphenoxide in xylene. The potassium salts of the mentioned acids **4a**, **4b** and **4c** are separated from the reaction mixture by treating with a 10% aqueous potassium hydroxide solution, because they have a good solubility in this solution and can be easily separated from xylene. The acids **2a**, **2b** and **2c** precipitate after treating the aqueous phase with a diluted hydrochloric acid solution.

The necessary phenoxides resulted from corresponding phenols and solid potassium hydroxide, in xylene, after the resulting water was removed by azeotropic distillation. The mentioned ketones were obtained through the cyclization of acids **2a**, **2b** and **2c**, applying two different working methods: (i) in poor yield by directly heating with polyphosphoric acids, and (ii) Friedel-Crafts cyclization of acid **5a**, **5b**, and **5c** chlorides, which were obtained by refluxing the corresponding acids with thionyl chloride in dry dichlorethane, the yields being better.

**Scheme 1:**
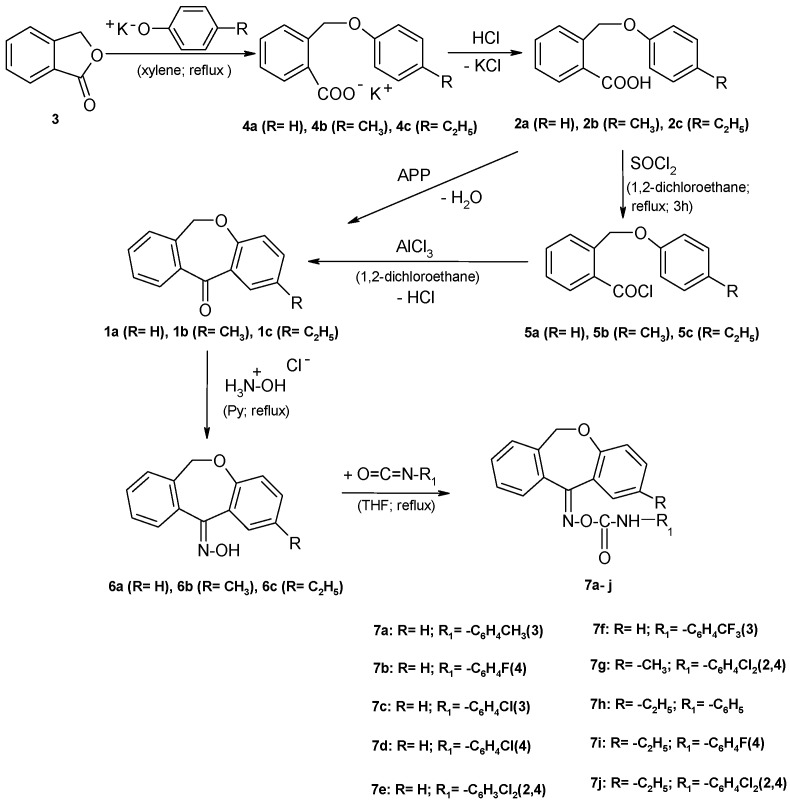
The pathway for the synthesis of the new dibenz[*b*,*e*]oxepins.

By refluxing the ketones **1a**, **1b**, and **1c** with hydroxilamine hydrochloride in pyridine gave oximes **6a**, **6b**, and **6c**. By treating these oximes with aryl isocyanates in anhydrous tetrahydrofurane yielded the final compounds **7a**–**j**. Scheme 1 shows the pathway for the synthesis of the new compounds.

The obtained compounds are white crystals, soluble in cold acetone and chloroform, soluble in hot inferior alcohols, ethyl acetate, benzene, toluene, DMSO. The structures of the new O-(arylcarbamoyl)-11-oximino-6,11-dihydro-dibenz[*b*,*e*]oxepins were established through IR and NMR spectroscopy. 

The IR spectra bands are described as w—weak band; m—medium band; s—intense band; vs.—very intense band and were obtained using the ATR technique. In the IR spectra some significant stretching bands due to νC=N and νN-O were observed at 1592–1631 cm^−1^ and 971–999 cm^−1^, respectively. The characteristic bands for the -CH_2_-O–moiety are: νCH_2_ sym: 2862–2885 cm^−1^; νCH_2_ asym: 2930–2982 cm^−1^; *δ*CH_2_ sym: 1301–1315 cm^−1^; δCH_2_ asym: 1456–1492 cm^−1^; νC-O-C sym: 999–1023 cm^−1^; νC-O-C asym: 1191–1219 cm^−1^. The IR also spectra revealed the presence of -O-C=O absorption bands as νC=O in the region 1728–1756 cm^−1^ and the νC-O at about 1218–1257 cm^−1^. The characteristic bands for the νNH of new compounds are 3259–3340 cm^−1^. The C-H aromatic absorption appear in the region 3068–3081 cm^−1^ and C=C at 1499–1594 cm^−1^. Halogen presence, in the molecules of new compounds, is proved by stretching bands situated at 1068–1102 cm^−1^ (for νC_ar_-F) and 744–797 cm^−1^ (for νC_ar_-Cl).

For recording NMR spectra the new dibenzoxepins were dissolved in CDCl_3_ and the chemical shift values, expressed in parts per million (ppm) were referenced downfield to tetramethylsilane, for ^1^H-NMR and ^13^C-NMR and the coupling constants (J) values are giving in Hertz. The chemical shifts for hydrogen and carbon atoms were established also by APT, GCOSY, GHMQC, and GHMBC, experiments. The ^1^H-NMR data are reported in the order: chemical shifts, multiplicity (s, singlet; d, doublet; dd, double doublet; ddd, doublet of double doublets; td, triple doublet; t, triplet; tt, triple triplet; q, quartet; m, multiplet; br, broad), the coupling constants, number of protons, and signal/atom attribution. For some compounds the signals attribution are the major (^M^) and minor (^m^) signals, produced by the *E*/*Z* isomerism.

The ^1^H-NMR spectra of the new dibenz[*b*,*e*]oxepins are divided into two spectral regions corresponding to the oxepinic system and to the phenylcarbamoyl group attached to the oximino group. The presence of an oxygen in the 5th position makes possible the existence of *E/Z* isomers, evident in our spectra through the splitting of the proton and the carbon signals. The difference between chemical shifts of methylene group is insignificant, but visible in the spectra as two singlets.

Analyzing the chemical shifts of the dibenz[*b*,*e*]oxepin scaffold in the ^1^H-NMR spectra, CH_2_O protons appeared as a singlet at 5.12–5.23 ppm as a minor signal and 5.17–5.22 ppm as a major signal in case a mixture of isomers was obtained or as a signal at 5.13–5.17 ppm in the case a single isomer was obtained. The hydrogen of the NH moiety appears in the 8.22–9.27 ppm range. The ^13^C-NMR spectra also displayed the characteristic signals of the suggested structures. The peak at about 155.64–158.36 ppm is attributed to C-11 and the signal of C-4a can be found in the 151.35–155.62 ppm range. 

The signal of the carbonyl carbon appears in the 157.52–161.54 ppm range. The methylene group (C-6) appears in the 70.49–70.56 ppm range as a major signal and 70.29–70.64 ppm as a minor signal and as a single signal at 70.52–70.70 ppm.

### 2.2. Bioinformatic Study of Compounds ***7a**–**j***

#### 2.2.1. Lipinski’s and Veber’s rule

Our results regarding the drug-like features showed that compounds **7a**–**j** respected Lipinski’s and Veber’s rules (Table 1). Lipinski’s rule of five states that a compound with a molecular mass under 500 Dalton (MW), a coefficient of partition between octanol and water (LogP_(o/w)_) lower than 5, no more than five hydrogen bond donors (HBD) and no more than 10 hydrogen bond acceptors (HBA) could be a good drug candidate. Veber’s rule says that a compound with 10 or fewer rotatable bonds (RTB) and a polar surface area (TPSA) no greater than 140 Ǻ^2^ should present good oral bioavailability.

#### 2.2.2. Predicted ADME-Tox

The compounds **7a**–**j** also have a very good predicted intestinal absorption, with values between 89–92% (Table 2), far higher than the threshold value of 30%, which predicts a good absorption through the small intestine [21]. The Caco-2 permeability values, calculated as logarithm of the apparent permeability coefficient (log Papp, cm/s), were in the range of 0.9 to 1.5, therefore higher that the threshold value of 0.9 predicting a good intestinal absorption (Table 2).

Regarding BBB permeability, it must be higher than 0.3 to consider that a compound will easily cross the BBB [21]. Thus, the tested compounds presented an average permeability, with the BBB permeability predicted values fluctuating between −0.3 and 0.2 (Table 2). The CNS permeability is predicted as the BBB permeability–surface area product (logPS). A compound with a predicted CNS permeability higher than–2logPS may penetrate the CNS [21]. All compounds **7a**–**j** are able to cross the CNS barrier, the recorded values in range of −1.2 and −1.6logPS indicating a high CNS permeability (Table 2).

Ames toxicity predicts the mutagenic potential of a compound [13]. Results from pkCSM platform shows that only **7a** compound presents Ames toxicity. The maximum tolerated dose for human (MTD) predicted for compounds **7a**–**j** is considered low (Table 3). Long QT syndrome is associated with inhibition of potassium channels encoded by hERG (I/II) gene [13]. PkCSM predictions indicate that compounds **7a**–**j** presented inhibitory activity on hERG II. LD50 represents the dose that will kill 50% of rats tested, in Table 3 being presented the LD50 prediction from both pkCSM and admetSAR platforms. LOAEL values representing the lowest dose of a compound that results in an observed adverse effect are presented in Table 3. Hepatotoxicity predictions indicate that compounds **7a**, **7c**, **7d** and **7h** are unlikely to disturb the liver normal function (Table 3). Both pkCSM and admetSAR platforms indicate that tested compound present *Tetrahymena pyriformis* toxicity. Regarding Minnow toxicity, the obtained results suggest that **7a** and **7h** compounds present Minnow toxicity. Carcinogenic effect prediction for molecules **7a**–**j** suggests that the molecules lack carcinogenic effects (Table 3).

### 2.3. Antimicrobial and Antibiofilm Activity of the Compounds ***7a**–**j***

The results of the qualitative assay of the antimicrobial activity of the tested compounds are listed in Table 4. The most active compound proved to be **7f**, which inhibited the microbial growth of four microbial strains, including Gram-positive (*B. subtilis*, *S. aureus*), Gram-negative (*E. coli*) bacterial and fungal strains (*C. albicans*). Compound **7a** exhibited anti-*B. subtilis* and anti-*C. albicans* activity, while the **7c** was active against *E. coli* and *C. albicans.* Compound **7b** exhibited a narrower antimicrobial spectrum, being active exclusively on *B. subtilis.* None of the tested compounds were active against *P. aeruginosa* and *K. pneumoniae* strains.

The quantitative assay results revealed a strong antimicrobial activity demonstrated by the low MIC values exhibited by **7a**, **7b** and **7f** against *B. subtilis* (4.8–39 μg/mL), by **7f** against *S. aureus* (4.8 μg/mL) and *E. coli* (78 μg/mL) and **7a** against *C. albicans* (4.8 μg/mL). In case of *B. subtilis* and *S. aureus*, the new compounds were more active than ticarcillin, the antibiotic used as positive control. The compound **7c** exhibited low antimicrobial activity (MIC 1250 μg/mL) against *E. coli* and *C. albicans* strains (Table 5).

The newly synthesized oxepines with the most intensive antibiofilm effect are those monosubstituted on the phenyl group. The compound **7c** exhibited the most intensive anti-fungal biofilm effect with an MBEC (minimum biofilm eradication concentration) of 4.8 μg/mL.

The newly synthesized oxepines **7a**, **7b** and **7f** exhibited an inhibitory effect on the adherence ability of *S. aureus* strain on a large scale of concentrations up to 39, 19.5 and 39 µg/mL, respectively. **7a** and **7f** inhibited the biofilm developed on the plastic substratum by *B. subtilis* strain up to 156 and 39 µg/mL, respectively. In both cases, the MBEC values were lower than those recorded for the antibiotic used as positive control. The high concentrations of **7a** (up to 625 µg/mL) inhibited the *E. coli* biofilm. The **7c** compound was extremely active against the fungal biofilm formed by *C. albicans* strain, up to the lowest tested concentration of 4.8 µg/mL, superior to that of fluconazole, followed by **7a** exhibiting anti-fungal biofilm activity up to 39 µg/mL (Table 6).

### 2.4. The Cytotoxicity of the Compounds ***7a**–**j***

**Figure 1:**
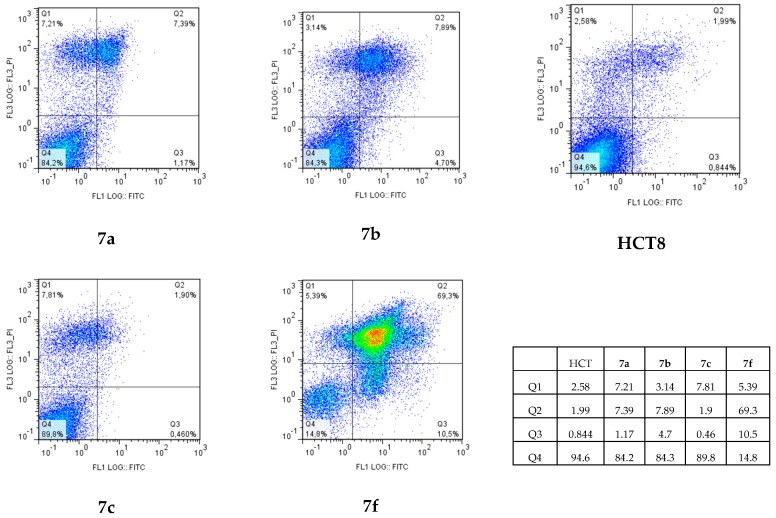
Analysis of apoptosis by flow cytometry. The dot plot figures show the following populations: necrotic (Q1—upper left), late apoptosis (Q2—upper right), early apoptosis (Q3—lower right), and viable cells (Q4—lower left).

The compounds with microbial activity were tested for toxicity. The compounds **7a**, **7b** and **7c** exhibited very low cytotoxicity levels on the HCT8 cells (the IC_50_ being 264.05, 251.93 and respectively 304.209), while the compound **7f** proved to be highly cytotoxic at the tested concentration of 100 µg/mL (IC_50_ = 54.269). Analysis by flow cytometry proved that apoptosis is the main mechanism induced after treatment with our compounds (Figure 1). 

## 3. Discussion

In a drug-repurposing-driven approach for speeding up the development of novel antimicrobial agents, this paper presents for the first time in the scientific literature the synthesis, physico-chemical characterization, in silico analysis, antimicrobial activity against bacterial and fungal strains in planktonic and biofilm growth state, as well as the in vitro cytotoxicity of new 6,11-dihydro-dibenz[*b*,*e*]oxepin-11(6H)one O-(arylcarbamoyl)oximes, obtained by treating some 11-hydroximino-6,11-dihydro-dibenz[*b*,*e*]oxepins with aryl isocyanates. We aimed to integrate in the same molecule the dibenzoxepinic nucleus and the biologically active oximino and phenylcarbamoyl groups. The structures of intermediary and final substances were confirmed through ^1^H-NMR and ^13^C-NMR spectra, IR spectra and by elemental analysis.

The bioevaluation started with in silico analyses allowing to predict some biopharmaceutical features of the obtained compounds. All the **7a**–**j** compounds respected Lipinski’s and Veber’s, predicting their good oral bioavailability. The predicted ADME-Tox indicated a very good intestinal absorption and good cellular permeability. 

All tested compounds have a high CNS permeability. AMES toxicity indicated a mutagenic potential only for the compound **7a**, however none of the tested compounds exhibit carcinogenic effects. The maximum tolerated dose predicted for humans was low for all the compounds. The toxicity could be related to the inhibition of hERG (I/II) potassium channels.

The antimicrobial activity of the tested compounds was performed on Gram-positive (*B. subtilis* ATCC 6633, *S. aureus* ATCC 25923), Gram-negative (*K. pneumoniae* 1771, *E. coli* ATCC 25922, *P. aeruginosa* ATCC 27853) bacterial, as well as *C. albicans* ATCC 10.231 fungal strains. 

In the qualitative assay the compounds bearing the methyl and ethyl groups in the position 2 of the dibenz[*b*,*e*]oxepin nucleus have been all inactive against any of the tested bacterial or fungal strain. Therefore, the quantitative assay was performed only for the compounds which proved to be active in the qualitative screening, respectively **7a**, **7b**, **7c** and **7f**. The tested compounds proved to be more active against the Gram-positive and fungal strains, the Gram-negative ones being more resistant, probably due to the increased impermeability of the outer membrane of these bacteria. 

The quantitative assay confirmed the qualitative screening results, revealing that the most active compound was **7f**. The -CF_3_ substituent could be related both to an intensive antimicrobial effect, demonstrated by the low MIC values and to a large antimicrobial spectrum, including Gram-positive and Gram-negative bacterial strains. The -CF_3_ has an intermediate electronegativity between -F and -Cl, being bioisosteric with -Cl and -CH_3_. It has been suggested that the -CF_3_ ketone derivatives are responsible for ATP synthesis decreasing, acting as an uncoupler when direct membrane damage might destroy the transmembrane proton gradient and consequently, the synthesis of ATP being inhibited. This action could explain the extended spectrum of antimicrobial activity, including the Gram-negative *E. coli* strain.

Concerning the potential relationship between the chemical structure and the antimicrobial activity, it is to be mentioned that the most active compounds, represented by **7f**, followed by **7a** are both substituted in the *meta* position of the benzene nucleus from the side chain. 

Among the genetic resistance of different microbial strains to numerous antimicrobial agents, a different type of phenotypic resistance, also called recalcitrance or tolerance, mediated by different mechanisms is occurring when microbial strains grow in biofilms [21]. The inert substrata including the prosthetic medical devices represent risk factors for the occurrence of biofilm associated infections. Giving the difficulty to treat biofilm associated infections, research is focusing on the discovery of novel strategies that block the adherence and biofilm development capacity [22,23,24]. The compounds active against different microbial strains in free state as suspended cells were further chosen and investigated concerning their efficiency against the adherent cells grown in biofilms developed in plastic wells. The experimental model uses mini volumes and multiple well plastic plates, allowing the simultaneous testing of a large spectrum of concentrations [25,26]. The tested compounds interacted differently with the biofilm development by different microbial strains. However, all compounds exhibiting anti-biofilm activity are mono-substituted with electron-withdrawing atoms or groups. The lowest MBEC value was obtained for **7c**, having a -Cl atom in *meta* position, followed by **7b**, with a -F atom in *para* and by 7**a**, with a methyl group in *meta* and **7f**, bearing a -CF_3_ group in *meta* position.

The microbicidal and antibiofilm compounds have been further investigated for their cytotoxicity and influence on the cell cycle phases. Annexin V binds early apoptotic cells that expose phospholipid phosphatidylserine (PS) from the inner to the outer membrane side. On the other hand necrotic cells that lack membrane integrity are stained with PI. The method allows for the ascertainment of typical features of dying cells and the loss of membrane integrity [27]. The mechanism of cell death seems to be the apoptosis in the case of compounds containing the F atom (**7b** and **7f**). The increasing the number of F atoms was associated with membrane integrity loss (**7f**) and rapid increase of secondary apoptosis, suggesting that compound **7f** has a great antitumor potential. However, in depth studies are needed in order to confirm this activity.

Overall, the generally low cytotoxicity of the tested compounds, excepting **7f**, could represent an advantage for the development of antimicrobial drugs with minimal side effects on the human cells and tissues.

## 4. Materials and Methods

### 4.1. General Information

All chemicals and solvents were purchased from Merck (Darmstadt, Germany), Fluka (Buchs, Switzerland), and Sigma–Aldrich (St. Louis, MO, USA) and were of commercial quality. They were used as received, except for 1,2-dichloroethane which was dried over calcium chloride and distilled at normal pressure, and pyridine which was stored over potassium hydroxide and then distilled. THF was dried by refluxing 1 h over potassium hydroxide and then distillated at normal pressure.

The structure, molecular formula, molecular weight, melting point, yield and elemental analyses of the new O-(arylcarbamoyl)-11-oximino-6,11-dihydro-dibenz[*b*,*e*]oxepins are presented in Table 7.

All melting points were recorded with an Electrothermal 9100 apparatus ((Bibby Scientific Ltd., Stone, UK) and are uncorrected. Elemental analyses were carried out using a CHNS/O Analyzer Series II 2400 apparatus (Perkin–Elmer, Waltham, MA, USA) and the results were within ±0.4% of theoretical values. IR spectra were recorded on a Vertex 70 FT–IR spectrophotometer (Bruker Corporation, Billerica, MA, USA). NMR spectra were recorded on a Unity Inova 400 instrument (Varian Medical Systems, Palo Alto, CA, USA) operating at 400 MHz for ^1^H and 100 MHz for ^13^C. New dibenz[*b*,*e*]oxepins were synthesized according to the previously described procedure [11,28,29].

### 4.2. General Procedure for the Synthesis of Novel Compounds

The final compounds were obtained after treating 0.01 mol 11-hydroximino-6,11-dihydro-dibenz[*b*,*e*]oxepin and its 2-methyl- and 2-ethyl-derivatives dissolved in 4 mL anhydrous THF with a solution of 0.01 mol of the corresponding aryl isocyanate in 1 mL anhydrous THF and refluxing in a round-bottom flask equipped with a condenser and drying tube. To obtain **7a**–**f** the reaction mixture was refluxed 55 h and for **7g**–**j** 80 h. Then the solvent was evaporated under reduced pressure. The resulting residue was purified from boiling ethyl acetate.

*6*,*11-Dihydrodibenz[b*,*e]oxepin-11(6H)one O-(3-tolyl-carbamoyl)oxime* (**7a**). ^1^H-NMR (CDCl_3_, δ ppm): 8.26 (br s, NHM); 8.20 (br s, NHm); 7.74 (dd, *J* = 1.7 Hz, *J* = 8.0 Hz, 1H, H-10); 6.91–7.60 (m, 11H, H-arom); 5.23 (s, H-6^M^); 5.17 (s, H-6^m^); 2.36 (s, CH_3_^m^) 2.30 (s, CH_3_^M^). ^13^C-NMR (CDCl_3_, δ ppm): 160.78 (C-12); 157.54 (C-11); 151.86 (C-4a); 138.10 (C-13); 136.78 (C-7a); 133.96 (C-15); 132.73 (C-3); 132.42 (C-10a); 130.59 (C-8); 130.36 (C-1); 129.98 (C-17); 128.95 (C-9); 128.44 (C-7); 128.35 (C-10); 121.36 (C-2); 120.35 (C-16); 119.82 (C-1a); 119.62(C-14); 117.45 (C-4); 116.85 (C-18); 70.64 (C-6^m^); 70.56 (C-6^M^); 21.48 (CH_3_^M^); 21.05 (CH_3_^m^). FT-IR (solid in ATR, ν cm^−1^): 3262m; 3149w; 3068w; 2982w; 2869w; 1730s; 1599s; 1558vs; 1529s; 1483m; 1442m; 1333w; 1301s; 1218vs; 1158m; 1108w; 1021s; 975vs; 942m; 903m; 801w; 774m; 751s; 691m; 635m; 583w; 514w; 442w.

*6*,*11-Dihydrodibenz[b*,*e]oxepin-11(6H)one O-(4-fluorophenyl-carbamoyl)oxime* (**7b**). ^1^H-NMR (CDCl_3_, δ ppm): 8.30 (br s, 1H, NH); 7.73 (dd, *J* = 1.7 Hz, *J* = 7.9 Hz, 1H, H-10); 7.35–7.62 (m, 6H, H-1, H-2, H-3, H-7, H-8, H-9); 7.06 (m, H-14, H-18); 7.04 (t, *J* = 8.5 Hz, 2H, H-15, H-17); 6.94 (dd, *J* = 1.3 Hz, *J* = 8.4 Hz, 1H, H-4); 5.16 (s, H-6^M^); 5.22 (s H-6^m^). ^13^C-NMR (CDCl_3_, δ ppm): 159.02 (*J* = 243.4 Hz, C-16); 157.52 (C-12); 156.2 (C-11); 152.04 (C-4a); 133.85 (C-13); 132.75 (C-3); 132.37 (C-7a); 130.95 (C-10a); 130.61 (C-8); 130.28 (C-1); 129.22 (C-9); 128.92 (C-7); 128.35 (C-10); 121.5 (C-14, C-18); 121.32 (C-2), 120.28 (C-4); 119.49 (C-1a); 115.78 (*J* = 22.8 Hz, C-15, C-17); 70.51 (C-6^M^); 70.29 (C-6^m^). FT-IR (solid in ATR, ν cm^−1^): 3268m; 3080w; 2930w; 2875w; 1740vs; 1601m; 1480w; 1464w; 1441m; 1423m; 1325s; 1305m; 1269m; 1250s; 1210s; 1161s; 1147s; 1133m; 1108w; 1077s; 1051m; 1006w; 981m; 846m; 752s; 731w; 707w; 675w; 631w; 548w.

*6*,*11-Dihydrodibenz[b*,*e]oxepin-11(6H)one O-(3-chlorophenyl-carbamoyl)oxime* (**7c**). ^1^H-NMR (CDCl_3_, δ ppm): 8.37 (br s, 1H, NH); 7.72 (dd, *J* = 1.9 Hz, *J* = 8.0 Hz, 1H, H-10); 7.58 (t, *J* = 1.9 Hz, 1H, H-14); 7.30–7.48 (m, 6H, H-1, H-2, H-3, H-7, H-8, H-9); 7.25 (t, *J* = 7.8 Hz, 1H, H-17); 6.94–7.15 (m, 2H, H-16, H-18); 6.92 (dd, *J* = 1.3 Hz, *J* = 8.4 Hz, 1H, H-4); 5.12 (s, H-6^M^); 5.20 (s H-6^m^)^13^C-NMR (CDCl_3_, δ ppm): 161.25 (C-12); 157.52 (C-11); 151.57 (C-4a); 138.16 (C-13); 134.74 (C-7a); 134.40 (C-15); 132.82 (C-16); 132.40 (C-10a); 130.65 (C-3); 130.32 (C-1); 130.09 (C-8); 129.24 (C-14); 128.88 (C-9); 128.36 (C-7); 127.95 (C-10); 126.91 (C-17); 121.62 (C-2); 119.66 (C-18); 119.42 (C-1a); 117.63 (C-4); 70.49 (C-6^M^); 70.29 (C-6^m^)FT-IR (solid in ATR, ν cm^−1^): 3271m; 3081w; 3001w; 2932w; 2900w; 2885w; 1740vs; 1592m; 1481s; 1461m; 1375w; 1342w; 1310m; 1283m; 1246vs; 1231s; 1216s; 1173m; 1155m; 1089m; 1065s; 1020s; 997vs; 919m; 8852w; 859m; 821m; 763m; 746s; 694w; 671w; 656w; 552w.

*6*,*11-Dihydrodibenz[b*,*e]oxepin-11(6H)one O-(4-chlorophenyl-carbamoyl)oxime* (**7d**). ^1^H-NMR (CDCl_3_, δ ppm): 8.30 (br s, 1H, HN); 7.71 (dd, *J* = 1.6 Hz, *J* = 7.7 Hz, 1H, H-10); 7.46 (d, *J* = 8.8 Hz, 2H, H-15, H-19); 7.31(d, *J* = 8.8 Hz, 2H, H-16, H-18); 7.28–7.50 (m, 5H, H-1, H-2, H-3, H-7, H-9); 7.01 (td, *J* = 1.4 Hz, *J* = 8.9 Hz, 1H, H-8); 6.95 (dd, *J* = 1.0 Hz, *J* = 8.5 Hz, 1H, H-4); 5.17 (s, 2H, H-6).^13^C-NMR (CDCl_3_, δ ppm): 161.18 (C-12); 157.58 (C-11); 151.71 (C-4a); 135.50 (C-13); 133.86 (C-7a); 132.85 (C-3); 132.42 (C-10a); 130.96 (C-16); 130.69 (C-8); 130.31 (C-1); 129.19 (C-14, C-18); 129.12 (C-9); 128.98 (C-7); 128.40 (CH-15, CH-17); 126.93 (C-10); 121.39 (C-2); 120.93 (C-1a); 120.35 (C-4); 70.56 (C-6).FT-IR (solid in ATR, ν cm^−1^): 3259s; 3193w; 3124w; 3069w; 2963w; 2904w; 2862w; 1733s; 1631m; 1598s; 1542vs; 1488vs; 1441m; 1399m; 1333w; 1306m; 1257m; 1208vs; 1155w; 1086s; 1045m; 1016vs; 974vs; 922w; 853w; 797s; 774m; 752m; 634m; 504m; 450w.

*6*,*11-Dihydrodibenz[b*,*e]oxepin-11(6H)one O-(2*,*4-dichlorophenyl-carbamoyl)oxime* (**7e**). ^1^H-NMR (CDCl_3_, δ ppm): 9.25 (s, 1H, NH); 8.24 (d, *J* = 8.8 Hz, 1H, H-18); 7.76 (dd, *J* = 1.8 Hz, *J* = 7.9 Hz, 1H, H-1); 7.59 (m, 1H, H-10); 7.41 (d, *J* = 2.4 Hz, 1H, H-15); 7.30–7.52 (m, 4H, H-2, H-7, H-8, H-9); 7.26 (dd, *J* = 2.4 Hz, *J* = 8.8 Hz, 1H, H-17); 7.03 (td, *J* = 1.2 Hz, *J* = 8.4 Hz, 1H, H-3); 6.94 (dd, *J* = 1.2 Hz, *J* = 8.4 Hz, 1H, H-4); 5.17 (s, H-6)^13^C-NMR (CDCl_3_, δ ppm): 161.34 (C-12); 157.90 (C-11); 151.35 (C-4a); 133.78 (C-13); 133.61 (C-7a); 132.79 (C-3); 130.72 (C-1); 130.65 (C-8); 130.12 (C-15); 129.21 (C-10a); 128.37 (C-9); 128.27 (C-7); 128.1 (C-10); 127.89 (C-17); 126.95 (C-16); 121.48 (C-14); 121.43 (C-2); 120.57 (C-18); 120.45 (C-4); 119.61 (C-1a); 70.70 (C-6)FT-IR (solid in ATR, ν cm^−1^): 3262m; 3072w; 2961m; 2930w; 2871w; 1746vs; 1604s; 1569m; 1477m; 1475w; 1439s; 1330m; 1305s; 1235vs; 1209s; 1181s; 1153m; 1112w; 1065vs; 1044vs; 1007s; 976vs; 889s; 851m; 797w; 744s; 695m; 630m; 547w.

*6*,*11-Dihydrodibenz[b*,*e]oxepin-11(6H)one O-(3-trifluoromethylphenyl-carbamoyl)oxime* (**7f**). ^1^H-NMR (CDCl_3_, δ ppm): 8.43 (br s, HN^M^); 8.39 (br s, HN^m^); 7.33–7.80 (m, 10H, H-arom); 7.06 (ddd, *J* = 1.2 Hz, *J* = 7.2 Hz, *J* = 8.4 Hz, 1H, H-2); 6.93 (dd, *J* = 1.2 Hz, *J* = 8.4 Hz, 1H, H-4); 5.22 (s, H-6^m^); 5.17(s, H-6^M^). ^13^C-NMR (CDCl_3_, δ ppm): 161.54 (C-12); 157.60 (C-11); 151.70 (C-4a); 137.56 (C-13); 136.78 (C-7a); 133.80 (C-15); 132.93 (C-3); 132.43 (C-10a); 130.75 (C-8); 130.32 (C-1); 129.77 (C-17); 129.31 (C-9); 128.67 (C-7); 128.43 (C-10); 122.74 (CF_3_); 121.43 (C-2); 121.24 (C-18); 120.91 (C-16); 120.37 (C-4); 119.40 (C-1a); 116.38 (C-14); 70.54 (C-6).FT-IR (solid in ATR, ν cm^−1^): 3267m; 3162w; 3105; 2964w; 2869w; 1740s; 1618m; 1564vs; 1492m; 1442m; 1335vs; 1310s; 1246m; 1204vs; 1161s; 1068s; 1023s; 982vs; 924w; 884s; 796m; 775m; 752m; 693m; 516w; 439w.

*2-Methyl-6*,*11-dihydro-dibenz[b*,*e]oxepin-11(6H)one O-(2*,*4-dichlorophenyl-carbamoyl)oxime* (**7g**). ^1^H-NMR (CDCl_3_, δ ppm): 9.22 (s, 1H, NH); 8.23 (d, *J* = 9.1 Hz, 1H, H-18); 7.58 (m, 2H, H-1, H-10); 7.42–7.51 (m, 3H, H-7, H-8, H-9); 7.42 (d, *J* = 2.3 Hz, 1H, H-15); 7.29 (dd, *J* = 2.3 Hz, *J* = 9.1 Hz, 1H, H-17); 7.19 (dd, *J* = 2.4 Hz, *J* = 8.4 Hz, 1H, H-3); 6.84 (d, *J* = 8.4 Hz, 1H, H-4); 5.14 (s, H-6); 2.33 (s, 3H, CH_3_)^13^C-NMR (CDCl_3_, δ ppm): 160.98 (C-12); 155.71 (C-11); 151.39 (C-4a); 133.73 (C-13); 133.41 (C-3); 132.87 (C-10a); 132.77 (C-2); 133.7 (C-7a); 130.76 (C-8); 130.69 (C-15); 130.04 (C-1); 128.98 (C-9); 128.83 (C-7); 128.4 (C-10); 128.35 (C-17); 128.08 (C-16); 123.52 (C-14); 121.11 (C-18); 120.18 (C-4); 118.86 (C-1a); 70.62 (C-6); 20.3 (CH_3_)FT-IR (solid in ATR, ν cm^−1^): 3271m; 3142w; 3089w; 3063w; 2964w; 2918w; 2869w; 1754vs; 1618w; 1565vs; 1491m; 1335s; 1310s; 1226s; 1161s; 1020s; 982vs; 775m; 752m; 742m; 516w.

*2-Ethyl-6*,*11-dihydro-dibenz[b*,*e]oxepin-11(6H)one O-(phenyl-carbamoyl)oxime* (**7h**). ^1^H-NMR (CDCl_3_, δ ppm): 8.22 (br s, 1H, NH); 7.59 (m, 1H, H-10); 7.53 (d, *J* = 2.4 Hz, 1H, H-1); 7.50 (dd, *J* = 1.3 Hz, *J* = 8.6 Hz, 2H, H-14, H-18); 7.49–7.39 (m, 3H, H-7, H-8, H-9); 7.35 (dd, *J* = 7.6 Hz, *J* = 8.6 Hz, 2H, H-15, H-17); 7.21 (dd, *J* = 2.4, *J* = 8.4 Hz, 1H, H-3); 7.12 (tt, *J* = 1.3 Hz, *J* = 7.6 Hz, 1H, H-16,); 6.85 (d, *J* = 8.4 Hz, 1H, H-4); 5.14 (s, 2H, H-6); 2.65 (q, *J* = 7.6 Hz, 2H, CH_2_-CH_3_); 1.26 (t, *J* = 7.6, 3H, CH_2_-CH_3_)^13^C-NMR (CDCl_3_, δ ppm): 161.21 (C-12); 155.64 (C-11); 151.85 (C-4a); 137.20 (C-13); 137.04 (C-7a); 134.06 (C-2); 132.56 (C-10a); 132.48 (C-3); 130.51 (C-8); 129.17 (C-15, C-17); 129.09 (C-1); 128.86 (C-10); 128.42 (C-9); 128.34 (C-7); 124.36 (C-16); 120.22 (C-4); 119.76 (C-14, C-18); 119.26 (C-1a); 70.55 (C-6); 27.85 (CH_2_-CH_3_); 15.65 (CH_2_-CH_3_)FT-IR (ATR in solid, ν cm^−1^): 3269m; 3198w; 3137w; 3081w; 2967w; 2932w; 2878w; 1728vs; 1601s; 1574vs; 1545s; 1499s; 1488s; 1415m; 1376w; 1326m; 1315s; 1302m; 1249m; 1204vs; 1196vs; 1151m; 1015m; 991s; 968s; 929m; 829w; 807m; 755s; 694m; 655m; 507w.

*2-Ethyl-6*,*11-dihydro-dibenz[b*,*e]oxepin-11(6H)one O-(4-fluorophenyl-carbamoyl)oxime* (**7i**). ^1^H-NMR (CDCl_3_, δ ppm): 8.24 (br s, HN); 7.58 (m, 1H, H-10); 7.52 (d, *J* = 2.4 Hz, 1H, H-1);7.45–7.35 (m, 3H, H-7, H-8, H-9); 7.22 (dd, *J* = 2.3, *J* = 8.6 Hz, 1H, H-3); 7.06 (m, 2H, H-14, H-18); 7.04 (t, *J* = 8.5 Hz, 2H, H-15, H-17); 6.86 (d, *J* = 8.4 Hz, 1H, H-4); 5.13 (s, 2H, H-6); 2.64 (q, *J* = 7.6 Hz, 2H, CH_2_-CH_3_); 1.25 (t, *J* = 7.6, 3H, CH_2_-CH_3_)^13^C-NMR (CDCl_3_, δ ppm): 161.4 (C-16); 160.79 (C-12); 158.36 (C-11); 155.62 (C-4a); 152.11 (C-13); 137.19 (C-7a); 133.99 (C-2); 132.54 (C-3); 132.51 (C-10a); 130.55 (C-1); 129.06 (C-8); 128.86 (C-9); 128.37 (C-7); 128.35 (C-10); 121.61 (C-14, C-18); 120.25 (C-4); 119.16 (C-1a); 115.89 (C-15, C-17); 70.52 (C-6); 27.84 (CH_2_-CH_3_); 15.67 (CH_2_-CH_3_)FT-IR (solid in ATR, ν cm^−1^): 3303m; 3077w; 2959w; 2931w; 2878w; 1731s; 1612m; 1521vs; 1456m; 1407m; 1376vs; 1303s; 1251m; 1191vs; 1156s; 1126s; 1102s; 999s; 957w; 920w; 874s; 836w; 812w; 769m; 698m; 641w; 619w; 551w; 519w.

*2-Ethyl-6,11-dihydro-dibenz[*b*,*e*]oxepin-11(6H)one O-(2,4-dichlorophenyl-carbamoyl)oxime* (**7j**). ^1^H-NMR (CDCl_3_, δ ppm): 9.27 (s, 1H, NH); 8.26 (d, *J* = 8.9 Hz, 1H, H-18); 7.59 (m, 1H, H-10); 7.53 (d, *J* = 2.4 Hz, 1H, H-1); 7.47–7.41 (m, 3H, H-7, H-8, H-9); 7.41 (d, *J* = 2.3 Hz, 1H, H-15); 7.29 (dd, *J* = 2.3 Hz, *J* = 8.9 Hz, 1H, H-17); 7.21 (dd, *J* = 2.3 Hz, *J* = 8.4 Hz, 1H, H-3); 6.85 (d, *J* = 8.6 Hz, 1H, H-4); 5.14 (s, 2H, H-6); 2.63 (q, *J* = 7.6 Hz, 2H, CH_2_-CH_3_); 1.25 (t, *J* = 7.6, 3H, CH_2_-CH_3_)^13^C-NMR (CDCl_3_, δ ppm): 161.12 (C-12); 155.95 (C-11); 151.44 (C-4a); 137.30 (C-13); 133.51 (C-7a); 132.97 (C-2); 132.85 (C-10a); 132.74 (C-3); 130.74 (C-1); 129.03 (C-16); 128.90 (C-8); 128.87 (C-9); 128.52 (C-7); 128.38 (C-10); 128.15 (C-17); 123.52 (C-14); 121.10 (C-18); 120.32 (C-4); 119.0 (C-1a); 70.7 (C-6); 27.92 (CH_2_-CH_3_); 15.67 (CH_2_-CH_3_)FT-IR (ATR in solid, ν cm^−1^): 3340m; 3110w; 2965w; 2931w; 2870w; 1756vs; 1618s; 1594vs; 1574w; 1503s; 1460s; 1412m; 1381w; 1315s; 1298m; 1249m; 1219vs; 1179vs; 1148m; 1126w; 1099m; 1057w; 1007w; 992s; 958s; 909m; 884w; 864w; 836w; 820m; 789w; 767s; 741w; 635m; 617m; 576w.

### 4.3. Bioinformatic Study of Compounds ***7a**–**j***

#### 4.3.1. Molecular Modeling

Molecular modeling of compounds **7a**–**j** was performed using the Discovery Studio software (Dassault Systèmes BIOVIA, San Diego, CA, USA, Discovery Studio Modeling Environment, Release 2017). In our study, the template molecular structure was doxepin. The spatial structures 3D for **7a**–**j** were obtained by using Build module of Discovery Studio. To minimize the energy of molecules we have used Hamiltoninan: Forcefield MMFF94x, at 0.05 gradient, after the minimisation partial charges Gasteiger (PEOE) have been applied. The refined 3D structures of **7a**–**j** were obtained as mol2 files.

#### 4.3.2. Determination of Drug–Like Character and Bioavailability

We have applied Lipinski’s rule of five to determine the drug-like activity and also, Veber’s rule to predict the bioavailability of those compounds. Lipinski’s rule of five states that a compound has drug–like activity if respects minimum 3 of the following criteria: a molecular mass less than 500 Da, a number of maximum of five hydrogen donors, a number of maximum 10 hydrogen acceptors and a partition coefficient between octanol and water (LogP_(o/w)_) smaller than 5 [28]. Veber’s rule states that a compound with a good oral bioavailability must have 10 or less rotatable bonds and the polar surface area should be less than 140 Å^2^ [29,30]. For performing the Lipinski and Veber rules, in Discovery Studio, we have calculated for the compounds **7a**–**j**, the molecular mass, number of maximum hydrogen donors/acceptor, LogP_(o/w)_, rotatable bonds and polar surface.

#### 4.2.3. Predicted Pharmacokinetic Profile of Compounds **7a**–**j**

In order to predict the pharmacokinetic profiles, ADME-Tox, of compounds **7a**–**j**, we have used the pkCSM platform [31]. For toxicity descriptors (e.g., Ames, carcinogenetic, hERGI/II inhibition, maximum tolerated dose (human), etc.) we used both pkCSMand also admetSAR platforms [32].

From the large number of predicted ADMET properties we have selected in this study: (i) intestinal absorption, (ii) Blood Brain Barrier (BBB) permeability, (iii) Central Nervous System (CNS) permeability, (iv) AMES toxicity (v) *Tetrahymena pyriformis* toxicity, (vi) Minnow toxicity, (vii) Rat lethal dose 50, (viii) hepatotoxicity, (ix) maximum tolerated dose (MTD), (x) human ether-a-go-go (hERG) I and II inhibition, (xi) the carcinogenic effect of a compound, (xii) Caco-2 permeability and (xiii) the lowest dose of a compound that results in an observed adverse effect (LOAEL).

### 4.4. Biological Assays

The antimicrobial activities of the synthesized compounds were determined against ATCC reference and clinical microbial strains, i.e., *Bacillus subtilis* ATCC 6633, *Staphylococcus aureus* ATCC 25923, *Escherichia coli* ATCC 25922, *Klebsiella pneumoniae* 350, *Pseudomonas aeruginosa* 1671 and *Candida albicans* ATCC 10231, using as positive controls the antibiotic ticarcillin and the antifungal agent fluconazole. Microbial suspensions of 1.5 × 10^8^ CFU/mL corresponding to 0.5 McFarland density obtained from 15–18 h bacterial cultures developed on solid media were used in our experiments. The antimicrobial activity was tested on Mueller-Hinton Agar (MHA) medium, while a Yeast Peptone Glucose (YPG) medium was used in case of *C. albicans*. The obtained compounds were solubilized in DMSO and the starting stock solution was of 5000 μg/mL concentration. The qualitative screening was performed by an adapted disk diffusion method as previously reported [33,34,35].

The quantitative assay of the antimicrobial activity was performed by the liquid medium microdilution method, in 96 multi-well plates, in order to establish the minimal inhibitory concentration (MIC). In this purpose, serial two-fold dilutions of the compounds ranging between 5000 and 4.8 µg/mL were performed in a 200 µL volume of broth and each well was seeded with 50 µL of microbial inoculum. Sterility control (wells containing only culture medium) and culture controls (wells containing culture medium seeded with the microbial inoculum) were used. The influence of the DMSO solvent was also quantified in a series of wells containing DMSO [36], diluted accordingly with the dilution scheme used for the compounds and for the positive controls. The plates were incubated for 24 h at 37 °C, and MIC values were considered as the lowest concentration of the tested compound that inhibited the visible growth of the microbial cultures incubated overnight. The visual determinations have been confirmed by absorbance measurements performed at 600 nm with an ELISA reader Apollo LB 911, the MIC value corresponding to an absorbance value significantly lower than that obtained for the culture control.

The assessment of the new compounds influence on the microbial ability to colonize the plastic inert substratum was performed by the micro-titer method, following previously described protocols. The absorbance at 490 nm was measured with an LB 911 ELISA reader (Apollo, Berthold Technologies GmbH & Co. KG, Bad Wildbad, Germany). All biological experiments were performed in triplicates [22,36,37].

### 4.5. Cytotoxicity

For the in vitro assessment of the cytotoxic effect, the HCT8 cells and the CellTiter Assay kit (Promega, Madison, WI, USA) and Annexin V-FITC Apoptosis Detection Kit I (BD Bioscience Pharmingen, Franklin Lakes, NJ, USA) were used. Briefly, for CellTiter Assay, 1 × 10^4^ cells/well were plated in 96 well plate and 24 h after, were treated with the new compound at final concentration between 400 µg/mL–10 µg/mL. The viability was evaluated after 24 h by adding CellTiter aqueous solution and reading to spectrophotometer according to the manufacturer indications. The IC50 were calculated from the percent of viability reported to untreated cells using on line tools https://www.aatbio.com/tools/ic50-calculator. For Annexin V-FITC Apoptosis Detection Kit I, 5 × 10^5^ cells were seeded in 3.5 cm diameter Petri dish and treated with 100 µg/mL compounds solution for 24 h. The total cells were resuspended in 100 µL of binding buffer, and stained with 5 µL Annexin V-FITC and 5 µL propidium iodide for 10 min in dark. At least 10,000 events from each sample were acquired using a flow cytometer (Beckman Coulter, Indianapolis, IN, USA). The percentage of cells affected by the treatment was determined by subtracting the percentage of apoptotic/necrotic cells in the untreated population from percentage of apoptotic cells in the treated population [38].

## 5. Conclusions

In the purpose of obtaining compounds with antimicrobial activity we have synthesized new dibenz[*b*,*e*]oxepin derivatives and performed chemical modulations in order to integrate in the same molecule the dibenzoxepinic nucleus and the biologically active oximino and phenylcarbamoyl groups. The new compounds were characterized by their main physical constants and ^1^H-NMR, ^13^C-NMR, and IR spectral analysis and by elemental analysis, which confirmed the structure of the obtained compounds.

The predicted values for the synthesized compounds **7a**–**j** suggested an optimal pharmacokinetic profile with an average lipophilic character that allows both cell membrane crossing and intestinal absorption.

The in vitro assay of the antimicrobial activity of the tested compounds showed that they exhibited a higher antimicrobial activity against the Gram-positive bacterial strains and *C. albicans*, representing thus potential candidates for the design of new therapeutic agents for microbial infections, especially those produced by multiresistant strains. The tested substances also inhibited the ability of *S. aureus*, *B. subtilis*, *E. coli* and *C. albicans* strains to colonize the inert substratum, accounting for their possible use as antibiofilm agents. All the active compounds exhibited very low cytotoxicity levels on the HCT8 cells, aspect that could represent an advantage for the development of new antimicrobial drugs with minimal side effects on the human cells and tissues, excepting the compound with high number of F that seems to have antitumor potential. 

## Figures and Tables

**Table 1:** Lipinski and Veber’s rules for compounds **7a**–**j**.

Compound	7a	7b	7c	7d	7e	7f	7g	7h	7i	7j
Rule										
**Lipinski’s Rule of Five**	yes	yes	yes	yes	yes (1 violation)	yes	yes (1 violation)	yes	yes	yes (1 violation)
HBA	4	5	4	4	4	7	4	4	5	4
HBD	1	1	1	1	1	1	1	1	1	1
MW(g/mol)	358.3	362.3	378.8	378.8	413.2	412.3	427.2	372.4	390.4	441.3
LogP_(o/w)_	4.16	4.11	4.36	4.35	5.11	4.86	5.26	4.47	4.78	5.57
**Veber’s Rule**	yes	yes	yes	yes	yes	yes	yes	yes	yes	yes
**RTB**	4	4	4	4	4	5	4	5	5	5
**TPSA(Å^2^)**	59.92	59.92	59.92	59.92	59.92	59.92	59.92	59.92	59.92	59.92

**Table 2:** Prediction of intestinal absorption, Caco-2, BBB and CNS permeability of compounds **7a**–**j**.

Compound	7a	7b	7c	7d	7e	7f	7g	7h	7i	7j	Unit
Descriptor											
**Intestinal Absorption**	92.1	92.7	92.3	91.8	89.7	90.9	89.9	90.9	92.2	89.2	% Absorbed
**Caco-2 Permeability PkCSM**	1.5	1.0	1.0	1.0	1.0	1.0	1.0	1.4	1.0	0.9	log Papp in 10^−6^ cm/s
**Caco-2 Permeability AdmetSAR**	1.5	1.6	1.6	1.6	1.6	1.5	1.6	1.3	1.4	1.5	LogPapp, cm/s
**BBB Permeability**	0.2	−0.0	−0.1	−0.0	0.0	−0.1	−0.3	0.2	−0.3	−0.2	log BB
**CNS Permeability**	−1.5	−1.6	−1.5	−1.5	−1.3	−1.4	−1.2	−1.5	−1.6	−1.3	log PS

**Table 3:** Toxicity predictions for compounds **7a**–**j**.

Compound	7a	7b	7c	7d	7e	7f	7g	7h	7i	7j	Unit.
Descriptor											
**Tox. AMES pkCSM**	yes	no	no	no	no	no	no	no	no	no	yes/no
**MTD**	0.14	0.04	0.07	0.08	−0.28	0.05	−0.32	0.28	0.01	−0.21	log mg/kg/zi
**hERGI Inhibition pkCSM**	no	no	no	no	no	no	no	no	no	no	yes/no
**hERGII Inhibition pkCSM**	yes	yes	yes	yes	yes	yes	yes	yes	yes	yes	yes/no
**LD50 pkCSM**	2.4	2.3	2.4	2.3	2.4	2.5	2.4	2.3	2.4	2.4	mol/kg
**LD50 admetSAR**	2.6	2.5	2.5	2.5	2.5	2.7	2.5	2.5	2.5	2.5	mol/kg
**LOAEL**	1.1	0.9	0.9	0.8	0.6	0.6	0.5	0.9	0.7	0.4	log mg/kg_bw/zi
**Hepatotoxicity**	no	yes	no	no	yes	yes	yes	no	yes	yes	yes/no
**Tox. *T. pyriformis* pkCSM**	0.3	0.3	0.3	0.3	0.3	0.3	0.4	0.3	0.3	0.3	log µg/L
**Tox. *T. pyriformis* admetSAR**	0.7	0.9	1.1	1.1	1.1	1.0	1.0	0.8	0.9	1.1	log µg/L
**Tox. Minnow pkCSM**	-0.9	0.8	0.1	0.5	0.1	0.3	−0.2	−0.9	0.7	−0.1	log mM
**Tox. Minnow admetSAR**	0.7	0.6	0.6	0.6	0.5	0.5	0.6	0.8	0.9	0.6	log mg/L
**Carcinogenic effect**	no	no	no	no	no	no	no	no	no	no	danger/warning/no

Tox.—toxicity, MTD—maximum tolerated dose.

**Table 4:** The results of the qualitative assay of the antimicrobial activity of the tested compounds by using an adapted diffusion assay (the growth inhibition diameters were measured and expressed in mm).

	Chemical Compounds	7a	7b	7c	7d	7e	7f	7g	7h	7i	7j	Antibiotic Positive Control (Ticarcillin)	Antifungal Positive Control (Fluconazole)
Microbial Strains													
***B. subtilis* ATCC 6633**		8 mm	7 mm	0	0	0	11 mm	0	0	0	0	15	-
***S. aureus* ATCC 25923**		0	0	0	0	0	8 mm	0	0	0	0	21	-
***P. aeruginosa* ATCC 27853**		0	0	0	0	0	0	0	0	0	0	17	-
***E. coli* ATCC 25922**		0	0	4 mm	0	0	10 mm	0	0	0	0	24	-
***K. pneumoniae* 1771**		0	0	0	0	0	0	0	0	0	0	0	-
***C. albicans* ATCC 10231**		8 mm	0	8 mm	0	0	12 mm	0	0	0	0	-	10

**Table 5:** The results of the quantitative assay of the antimicrobial activity of the compounds selected by the qualitative screening and the corresponding MIC value (μg/mL).

	Chemical Compounds	7a	7b	7c	7f	Antibiotic Positive Control (Ticarcillin)	Antifungal Positive Control (Fluconazole)
Microbial Strains							
***B*. *subtilis* ATCC 6633**		4.8	4.8	>5000	39	78	-
***S. aureus* ATCC 25923**		>5000	>5000	>5000	4.8	9.76	-
***E. coli* ATCC 25922**		>5000	>5000	1250	78	19.5	-
***C. albicans* ATCC 10231**		4.8	>5000	1250	-	-	4.8

**Table 6:** The results of the quantitative assay of the antibiofilm activity of the compounds selected by the qualitative screening and the corresponding MBEC value (μg/mL).

	Chemical Compounds	7a	7b	7c	7f	Antibiotic Positive Control (Ticarcillin)	Antifungal Positive Control (Fluconazole)
Microbial Strains							
***B*. *subtilis* ATCC 6633**		156	-	-	39	156	-
***S. aureus* ATCC 25923**		39	19.5	-	39	156	-
***E. coli* ATCC 25922**		625	-	-	-	625	-
***C. albicans* ATCC 10231**		39	-	4.8	-	-	39

**Table 7:** Characterization data of the new compounds.

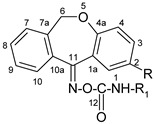 **7a–j**												
No	R	R_1_	Molecular Formula	Mol. Weight	Melting Point (°C)	Yields (%)	C%		H%		N%	
							c	e	c	e	c	e
**7a**	-H	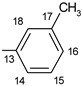	C_22_H_18_N_2_O_3_	358.38	131.6–132.9	76	73.73	73.41	5.06	5.14	7.82	7.82
**7b**	-H	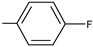	C_21_H_15_FN_2_O_3_	362.35	189.5–193.1	72	69.60	69.41	4.17	4.17	7.73	7.70
**7c**	-H	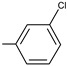	C_21_H_15_ClN_2_O_3_	378.81	175.3–178.4	67	66.58	66.43	3.99	3.91	7.39	7.28
**7d**	-H	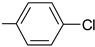	C_21_H_15_ClN_2_O_3_	378.81	165.2–167.4	64	66.58	66.24	3.99	4.14	7.40	7.47
**7e**	-H	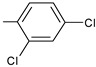	C_21_H_14_Cl_2_N_2_O_3_	413.25	181.2–183.3	54	61.03	61.31	3.41	3.45	6.78	6.69
**7f**	-H	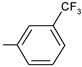	C_22_H_15_F_3_N_2_O_3_	412.36	164.2–165.8	79	64.08	63.77	3.67	3.79	6.80	6.69
**7g**	-CH_3_	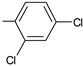	C_22_H_16_Cl_2_N_2_O_3_	427.28	150.4–152.7	59	61.83	61.97	3.77	3.69	6.55	6.49
**7h**	-C_2_H_5_	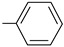	C_23_H_20_N_2_O_3_	372.42	173.7–175.9	71	74.18	74.34	5.41	5.32	7.52	7.38
**7i**	-C_2_H_5_	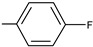	C_23_H_19_FN_2_O_3_	390.4	178.2–181	73	70.76	70.51	4.91	4.87	7.18	7.17
**7j**	-C_2_H_5_	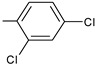	C_23_H_18_Cl_2_N_2_O_3_	441.3	192–194.1	69	62.59	62.78	4.11	4.02	6.35	6.39
